# Automated Electrical Detection of Proteins for Oral Squamous Cell Carcinoma in an Integrated Microfluidic Chip Using Multi-Frequency Impedance Cytometry and Machine Learning

**DOI:** 10.3390/s25051566

**Published:** 2025-03-04

**Authors:** Muhammad Tayyab, Zhongtian Lin, Seyed Reza Mahmoodi, Mehdi Javanmard

**Affiliations:** 1 Department of Electrical and Computer Engineering, Rutgers University, Piscataway, NJ 08854, USA; mt1013@scarletmail.rutgers.edu (M.T.); zl143@scarletmail.rutgers.edu (Z.L.); 2 Department of Electrical and Computer Engineering, University of Denver, Denver, CO 80210, USA; seyedreza.mahmoodi@du.edu

**Keywords:** biosensors, microfluidics

## Abstract

Proteins can act as suitable biomarkers for the prognosis and diagnosis of certain conditions and can help us gain an understanding of the fundamental processes that occur inside an organism. In this work, we present a fully automated machine learning-assisted label-free method for the electrical detection of proteins in an integrated microfluidic chip using multi-frequency impedance cytometry and off-the-shelf components for realizing an automated and programmable fluid control system. We verify the robustness of our mixing method on our custom microfluidic mixer composed of polydimethylsiloxane (PDMS) serpentine channels optically using a fluorescent sandwich immunoassay and comparing the results with a commercial benchtop mixer. Salivary IL-6 is a biomarker for oral squamous cell carcinoma (OSCC), and we have demonstrated that our system can be used for the detection of quantification of Interleukin-6 (IL-6) levels in a solution using the impedance response of beads conjugated with the protein of interest, which passes through the microfluidic chip with reasonable accuracy (96%). Although we have demonstrated the detection and quantification of IL-6, our system can be adapted to any protein of interest with slight modification in the reagents and bead-binding protocols.

## 1. Introduction

Proteins are an essential component and building block of the human body. These large and complex molecules are composed of long chains of amino acids and regulate the function and operation of various processes [1]. Proteins have a wide range of applications inside the human body, ranging from regulating gene expression [2] to the crucial steps for the formation and the deletion of cells, i.e., apoptosis [3]. Therefore, by accurately detecting and quantifying proteins, we can gain an understanding of the processes occurring in the human body and can potentially predict and manage life-threatening diseases such as cancer [4]. Biomarkers play an important role in the diagnosis and prognosis of various diseases. A biomarker is defined by the World Health Organization (WHO) as “any substance, structure, or process that can be measured in the body or its products and influence or predict the incidence of outcome or disease” [5]. Proteins can thus be used as effective biomarkers for the prediction and prevention of various diseases such as viral infections [6], inflammation [7], and even early detection of cancer [8].

According to the Centers for Disease Control and Prevention (CDC), in the United States, in 2019, 1,752,735 new cancer cases were reported, and 599,589 people died of cancer [9]. Furthermore, studies indicate that the diagnosis of cancer in the early stages gives patients a better chance of survival through chemotherapy and other exploratory treatments [10]. Squamous cell carcinoma accounts for 90% of all oral cancers. This type of cancer may be present in any part of the mouth but is generally found to affect the tongue and the floor of the mouth [11]. This type of cancer predominantly occurs in populations with known risk factors, such as tobacco and alcohol use [12]. Saliva is an oral fluid produced in the mouth that can play a vital role in the early detection of oral squamous cell carcinoma. Researchers studied several salivary protein biomarkers that can be used for the detection of oral squamous cell carcinomas, such as Interleukin-6 (IL-6), Interleukin-8 (IL-8), and tumor necrosis factor-alpha (TNF-α), to name a few [13]. They found significant differences between the concentrations of the salivary biomarkers in the cancer patients’ group and the salivary biomarker concentrations in the control group.

One proven way of measuring proteins and antibodies is the predominantly used traditional techniques of Enzyme-Linked Immunosorbent Assay (ELISA), which continues to be the gold standard for protein and antibody detection [14]. In a traditional ELISA technique, an enzyme, usually in the solid phase, is used to detect the presence of a ligand, usually in a liquid sample, using antibodies that are specific to the protein to be measured. The procedure is typically performed on a 96-well plate [15]. Although the traditional ELISA technique provides great accuracy and remains the benchmark for protein detection, there are limitations to its use in its traditional 96-well plate format. As an example, ELISA requires a skilled technician for its use, long incubation times, and a large volume of reagents, making its use in a resource-limited setting difficult and costly [16].

Microfluidics, which is the manipulation of fluids in microfluidic channels, i.e., channels with dimensions in the range of tens to hundreds of micrometers, has emerged as a new field in the past couple of decades to address the challenges associated with various biological and chemical assays [17]. Microfluidic-based assays for protein detection offer potential alternative solutions to the problems that plague the traditional ELISA-based systems [18,19,20]. When considering microfluidic immunoassays and immunosensors, there is a wide variety of sensing modalities to choose from, which can be broadly classified into optical/fluorescent [21], colorimetric [22], and electrochemical-based detection [23]. Each of the sensing modalities has certain advantages and disadvantages associated with the technique, and the choice of the sensing modality used will depend on factors such as the required accuracy, cost, and complexity [18]. Electrical-based detection offers an inherent advantage due to the miniaturization of components, thereby reducing costs and making the system portable and easy to use [24,25,26]. Moreover, with the advent of artificial intelligence (AI) and machine learning (ML), we can apply these techniques to the data obtained from microfluidic systems for the automated detection of various analytes of interest and particularly for the detection of proteins [27,28].

In this paper, we present a machine learning-assisted label-free method for the electronic detection of proteins in an integrated microfluidic chip and demonstrate the automation of our method by using machine learning in conjunction with an automated fluid control system utilizing off-the-shelf components, thereby reducing the complexity for performing the immunoassay [29]. The integrated microfluidic chip is composed of two parts: (1) the mixer composed of serpentine channels and (2) a microfluidic pore for the passage of beads over integrated gold electrodes on a glass wafer. The automated fluid control system is composed of three main off-the-shelf components: (1) reservoirs for holding the fluid and reagents, (2) a programmable syringe pump, and (3) a programmable 3-port selector valve. We use this automated fluid control system for interfacing the microfluidic chip with our sample of interest, which is mixed with the protein antibody soup. Once the fluid is mixed in the microfluidic chamber composed of serpentine channels, we then use an impedance spectroscope interfaced with the integrated gold electrodes to record the impedance data at eight different frequencies. This data is analyzed through the developed ML models for the detection and quantification of proteins. It has been established that salivary IL-6 can be used as a biomarker for the detection of oral squamous cell carcinoma [13]. Therefore, we chose IL-6 as a candidate for demonstrating the detection of proteins since the goal of this study is to inch us closer to the point-of-care detection of oral squamous cell carcinoma, although the system may be adapted for any protein of interest.

## 2. Materials and Methods

### 2.1. Bead-Binding Protocol

The process of binding beads consists of two phases. The M-280 Tosyl-activated beads must first be washed. Pairing the antibodies is the next stage. We transfer 10 µL of beads to a tube for the washing process. Then, 200 µL of buffer B (0.1 M Na-phosphate buffer, pH 7.4) is added. The supernatant is then removed from the tube and put in a magnet. The beads are then resuspended in 10 µL of buffer B once the tube has been withdrawn from the magnet. The washing process comes to an end here. The next stage is coupling, where the beads and antibodies are joined together.

In order to do this, we move the beads into a fresh tube and affix the tube to a magnet. The supernatant is then eliminated. The beads are then resuspended in 5 µL of anti-IL-6 antibody after the supernatant has been removed. After that, we add buffer B, bringing the total volume to 40 µL. Following that, we pipette or vortex mix 30 µL of buffer C (3 M ammonium sulfate in buffer B). The soup is then incubated in the shaker for 18 to 24 h at 37 °C. We put the tube in the magnet and extract the supernatant after 24 h. After that, the tube is taken out of the magnet, and 200 µL of buffer D (PBS pH 7.4 with 0.5% (*w*/*v*) BSA) is poured over the beads. After that, the mixture is shaken for an hour at 37 °C. Following incubation, we magnetize the tube and extract the supernatant. Then, 200 µL of buffer E (PBS pH 7.4 with 0.1% (*w*/*v*) BSA) is added once the tube has been taken out of the magnet. The process is repeated, this time adding 200 µL of buffer E and collecting the supernatant inside the magnet. To acquire the necessary concentration for the experiment, the beads are lastly diluted using buffer E.

### 2.2. Microfluidic Chip Fabrication

The microfluidic chip has embedded gold electrodes and is built of PDMS on a glass surface. Patterning and creating the electrodes on the glass wafer is the initial stage in creating the microfluidic chip. A 3″ fused silica wafer is used to manufacture electrodes on glass using conventional photolithography. The steps in the procedure include liftoff processing, electron beam metal evaporation, and photo-patterning resistance on the fused silica wafer. Wafer cleaning, spin coating of the photoresist, soft baking of the resist, exposure to ultraviolet light via a chromium mask printed on a 4-by-4 glass plate, development of the resist, and hard baking of the resist are all steps in the photo-patterning process. After photo-patterning, an electron beam evaporation technique deposits a 100-nm-thick coating of gold on the substrate. For better gold adherence to the glass wafer, a 10-nm coating of chromium is utilized; otherwise, the gold film is readily torn off. Due to its inertness and resistance to corrosion, gold was selected as the electrode. The electrodes were 20 µm wide, with a 15 µm gap between each electrode.

Using soft lithography, we created the mixer chip itself and the microfluidic channel out of polydimethylsiloxane (PDMS). On a 3″ silicon wafer that serves as a master mold, a layer of SU-8 was patterned. Standard cleaning, spin coating, soft baking, exposure, development, and hard baking are all steps in the SU-8 photo-patterning process. Following the creation of the master mold, PDMS (10:1 prepolymer/curing agent) was applied to the master mold and baked for two hours at 80 °C to cure it. Then, the PDMS channel was separated from the mold. The entrance and outflow were then formed by punching two holes, one measuring 5 mm and the other 3 mm. After both substrates had received oxygen plasma treatment, the PDMS substrate was then positioned and adhered to the electrode chip. The irreversible bond was then created by baking the chip for 40 min at 70 °C. Our microfluidic channel was 20 µm wide and 15 µm tall.

### 2.3. Electrical Parameters

Zurich Instruments’ HF2IS (Zurich, Switzerland) commercial benchtop impedance spectroscope was used to record the electrical readings. A minimum of 25 min was spent recording each measurement. A variety of electrical settings on the commercial benchtop impedance spectroscope may be adjusted for different applications. Up to eight distinct frequency excitation sources can be generated and demodulated concurrently. We used each of the eight distinct frequency-generating and demodulation capabilities for the studies. The precise frequencies utilized were 500 kHz, 700 kHz, 1 MHz, 1.5 MHz, 2 MHz, 5 MHz, 10 MHz, and 20 MHz. We chose 400 mV as the excitation voltage and 1 kV/A as the trans-impedance amplification factor for each of the frequencies. For the purpose of recording using the integrated Analog-to-Digital Converter (ADC) incorporated in the commercial benchtop impedance spectroscope, the trans-impedance amplifier transforms the current signal taken from the electrode and converts it into a voltage signal. We found that a single bead needs at least 10 ms to traverse the 15 µm gap between the two gold electrodes, which serves as the sensing area of the microfluidic chip’s electrical detection region. As a result, we chose a bandwidth of 100 Hz for the Impedance Spectroscope’s Low Pass Filter. This implies that any particle traveling across the sensing zone quicker than 10 ms or any change in impedance that occurs more quickly than 10 ms, such as jitter, would be filtered out of the signal.

### 2.4. Experimental Protocol

LabSmith (Livermore, CA, USA) off-the-shelf components are used for making a fully automated fluid control system [29]. The reservoir is filled with 120 µL of beads, which are already functionalized with the anti-IL-6 antibody. The sample to be tested is loaded into the breadboard reservoir (BBRES-1 mL—T116, LabSmith). We only need about 20 µL of sample for the sensing region; however, there is some dead volume associated with mixing that has to be accounted for. Therefore, we load 50 µL of the sample solution into the reservoir. The sample solution is either PBS, which is used as the control group, or IL-6 protein, which has been reconstituted in PBS to give the required concentration. The programmable syringe pump (SPS01-080—T116, LabSmith) is then programmed to take out the solution from the breadboard reservoir into the syringe pump by controlling the 3-port microprocess valve. This fluid is then pumped into the microfluidic chip. This process is repeated twice so that the mixer is completely filled up with the solution and the fluid can reach the electrical sensing region of the microfluidic chip. Once the fluid starts flowing through the sensing region, we record the electrical impedance data using the Zurich Instruments HF2IS impedance spectroscope. Each experiment is allowed to run for at least 25 min; typically, around 30 min of data is recorded. It is important to note that we did not use the automated fluid control system for carrying out all the experiments in order to save time due to the two pumping steps required. Therefore, once we verified that we could do the experiments using the automated system, we used a syringe to take the fluid out from the reservoir and pump it into the microfluidic chip. This data is saved to a Personal Computer via a USB port that is connected to the commercial benchtop impedance spectroscope. The data analysis is done offline to train and test the ML models. We did the experiments on two different chips and used five different concentrations of IL-6 for chip 1: 0 pg/mL (negative control), 10 pg/mL, 50 pg/mL, 100 pg/mL, and 500 pg/mL. For chip 2, we also recorded the data for two additional concentrations: 25 pg/mL and 300 pg/mL. Although these additional concentrations were not used in this work, we plan to develop a regression model in the future using the larger dataset by adding more concentrations.

### 2.5. Machine Learning Framework

The machine learning models were developed using MATLAB’s classification learner (MathWorks MATLAB, R2021b (Natick, MA, USA)). The machine learning framework is explained in detail here for both the protein quantification and the detection of oral squamous cell carcinoma, i.e., the binary classifier. When a bead passes over the gold electrodes, there is a momentary change in the impedance of the system due to a change in the solution resistance and the solution capacitance values. This is recorded as a sudden spike or a peak in the recorded impedance data. We note the peak amplitude value of these peaks and store each of these peaks in a matrix where each peak has eight different data points due to the eight simultaneous excitation frequencies used. These peaks are then split into a training and test dataset using a 70:30 ratio, respectively. The training dataset is further divided into a 70:30 ratio, such that 30% of the peaks are used for training layer 1 of the ML model, which is a model developed for individual beads, whereas 70% of the dataset is used for training layer 2 of the developed ML model, which is a model for the whole sample instead of the individual peak data. The layer 2 training data is also divided randomly into 100 mock experiments so that the training data can correspond to a sample volume and an assay time much smaller than 30 min, i.e., approximately 3 min worth of data. These steps are the same for both the protein quantification and the binary classifier ML models. For the protein quantification, we use a coarse k-nearest neighbor algorithm for both layer 1 and layer 2 of the ML models. For the binary classifier, we label all the other concentrations except the negative control group as a protein group. Furthermore, we also pool the data from two chips to develop the binary classifier. The protein group is much larger than the negative control group; therefore, we randomly select peaks from this dataset such that the sizes of both the control group and the protein group are comparable. For the binary classifier, we found that the linear discriminant model yielded the best results for layer 1 of the ML model, whereas the coarse k-nearest neighbor proved to be the best choice for layer 2 of the ML model. These models were applied to the test data that we had separated at the beginning of the analysis. The test data is also divided into “mock experiments” such that 10% of the peaks are selected from each concentration group. This is done in order to verify that our algorithm can work with smaller datasets which correspond to lower sample volumes and less assay time, i.e., approximately 3 min.

## 3. Results

### 3.1. System Overview

**Figure 1:**
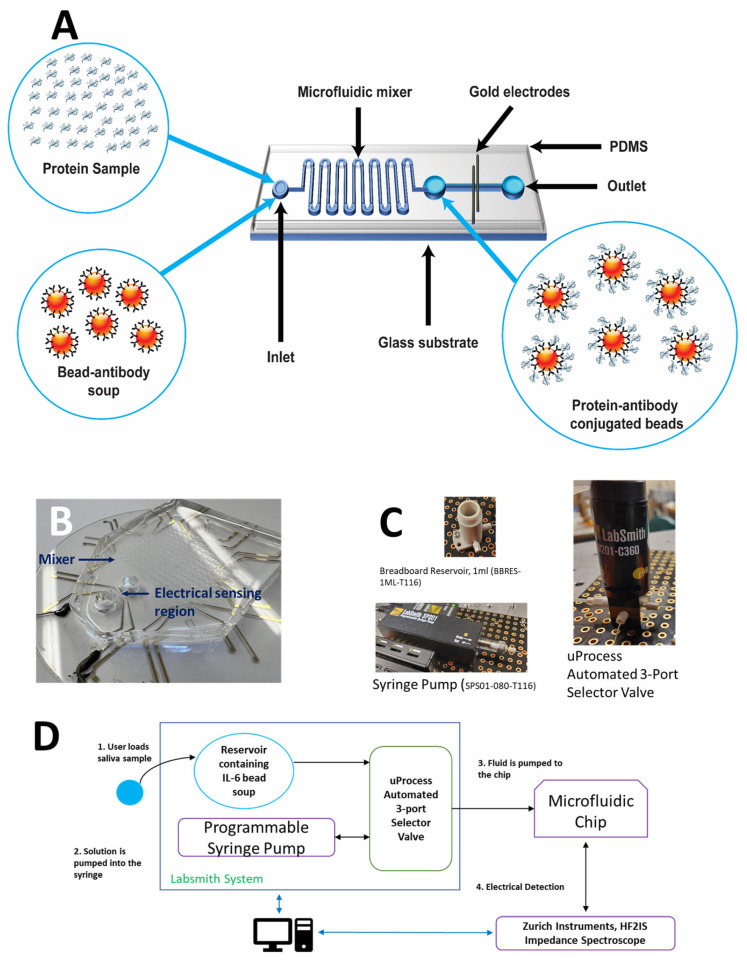
System Overview. (**A**) Simplified diagram depicting the principle of operation for the electronic detection of protein antibody-conjugated beads. (**B**) Photograph of the integrated microfluidic chip consisting of the mixer and the electrical detection pore over the gold electrodes. (**C**) Commercial off-the-shelf components that make up the automated fluid control system—LabSmith, Inc. [29]. (**D**) Process flow diagram demonstrating the bird’s eye view of the step-by-step protocol for the automated detection of proteins using the automated system made up of commercial off-the-shelf components.

With the aid of Machine Learning (ML) methods on data obtained through multi-frequency impedance spectroscopy, we demonstrate an automated label-free method for protein detection and quantification in a reusable microfluidic chip. The simplified diagram in Figure 1A, which shows the protein detection system’s operating principle, gives a system summary. As shown in the figure, we begin by taking functionalized 2.8 µm magnetic beads that have undergone a surface modification procedure to add antibodies to the target protein to the surface. In a microfluidic mixing chamber, these beads are combined with the sample containing the target proteins for the identification and quantification of the target protein. Serpentine channels are used in the microfluidic mixing container to speed up the mixing. The bead protein soup is transferred to the microfluidic chip with built-in gold electrodes after passing through the mixing chamber for the purpose of electrically detecting the beads. The space between the gold electrodes serves as the sensing zone for the beads. The electrodes were patterned on a glass wafer using conventional lithography methods. Figure 1B shows the glass wafer with the patterned gold electrodes with the polydimethylsiloxane (PDMS) mixer chip bonded on top of the gold electrodes. In a possible future iteration of the chip, the two electrode pairs on a microfluidic polydimethylsiloxane (PDMS) channel grant us the capability for prospective multiplexed sensing of analytes and proteins. Figure 1C shows the major components of the programmable fluid control system used for automating the process of the detection of proteins. We have demonstrated that this system can be deployed for the sensing of proteins with minimal input from the user, as the user only needs to load a sample into the reservoir for the purpose of detection and quantification of the proteins. The system consists of a syringe pump, a 3-port valve, and a reservoir containing the bead-antibody soup to which the user adds the sample.

Figure 1D demonstrates the step-by-step process for the detection of proteins. In this process, the user starts out by placing the sample, which has to be processed for the detection of a target protein, into the reservoir. The 3-port valve is programmed to be configured in such a way that the fluid is extracted from the syringe from the reservoir. Once this step has been completed and the syringe is filled with the fluid, the valve is turned to the alternative position so that fluid can be pumped into the microfluidic chip. We can then perform electrical detection of the proteins using the impedance spectroscope and process the data using the Machine Learning (ML) models for the specific protein.

### 3.2. Microfluidic Mixer Characterization Using IL-6 Sandwich Immunoassay

**Figure 2:**
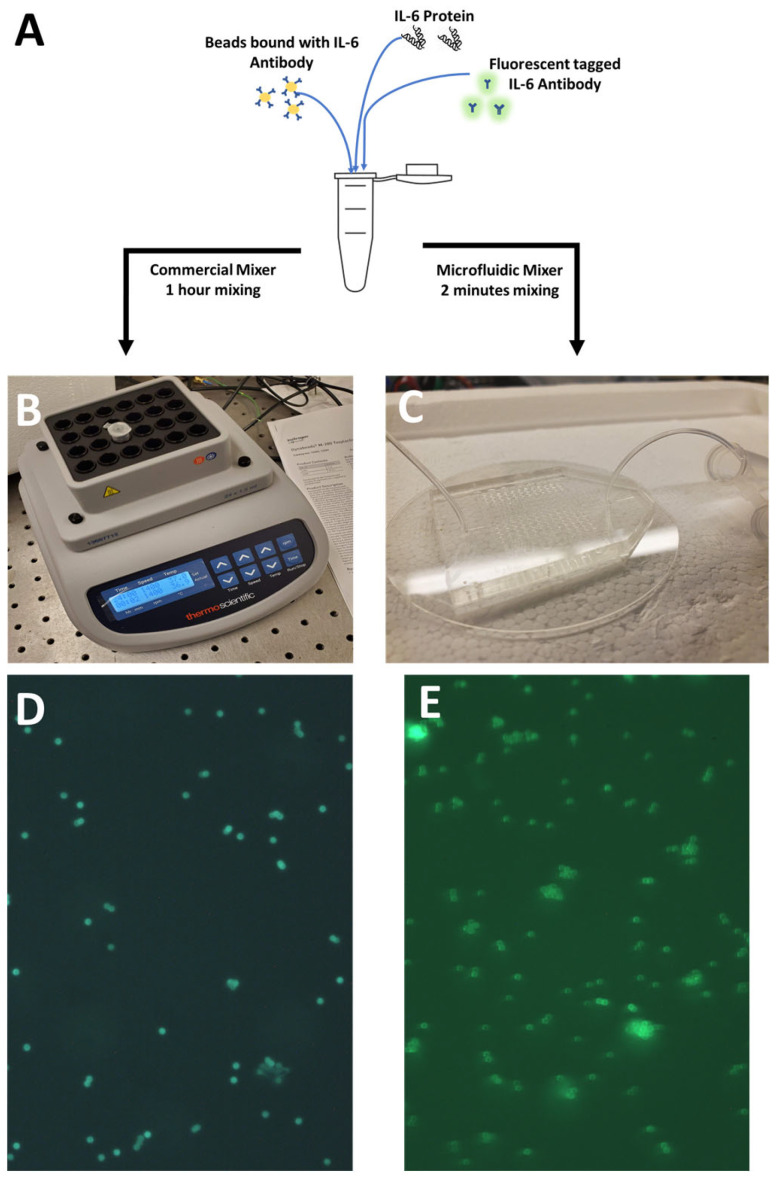
Verifying robustness of the protein-bead binding protocol. (**A**) Sandwich immunoassay for verification of the bead-binding protocol for IL-6. (**B**) Commercial benchtop mixer. (**C**) Microfluidic mixer with serpentine channels used for mixing the protein-bead soup. (**D**) Microscopic image for the sandwich assay with mixing of IL-6 protein bead antibody soup performed on a benchtop commercial mixer for 1 h. (**E**) Microscopic image for the sandwich assay with mixing of IL-6 protein bead antibody soup performed on the microfluidic mixer with serpentine channels for 2 min.

The sandwich immunoassay is one of the most widely used techniques for protein identification. This approach relies on a protein’s precise attachment to two antibodies. The second antibody is conjugated to a sensing component, such as a nanoparticle or bead, while the first antibody is connected to a fluorescent dye or a chemiluminescent substrate. The proteins in the sample bind to the antibody-dye or antibody-substrate combination when it is injected into a mixer, producing a detectable signal. Additional chemicals, such as the secondary fluorescent antibody, and optical devices, such as a microscope, are needed for this technique of protein detection in order to identify it. We carried out a test to confirm the reliability of our bead antibody protocol and the mixing methodology. In addition, we contrasted the fluorescence of a commercial benchtop mixer with the mixing chamber designed specifically for microfluidics. The various parts of the sandwich immunoassay are shown in Figure 2A. For this ELISA, the protein Interleukin-6 (IL-6) served as the target protein. The protein antibody bead soup with the extra fluorescent tag was divided into two equal portions using the same set of reagents. The commercial benchtop mixer was used to combine one component, and the custom microfluidic chip with serpentine passages was used to combine the other. In Figure 2B, a professional benchtop mixer is depicted. The custom microfluidic mixing container experimental setup can be seen in Figure 2C. A syringe is used to regulate the flow in the mixer so that it takes the fluid 2 min to travel its entire length. After that, the fluid is gathered inside an Eppendorf tube so that it can be seen under a bright microscope. The material is similarly taken from the commercial benchtop mixer after an hour of mixing so that it can be imaged with a fluorescent microscope. Figure 2D and Figure 2E, respectively, show the mixing pictures for the custom mixing chamber and the commercial benchtop mixer. It is significant to note that the benchtop mixer required more than an hour to mix to produce the outcome seen, whereas the custom microfluidic chip required just two minutes. We incorporate tesla valves, which are microstructures that allow fluid movement in only one direction due to their shape, in our custom microfluidic mixer to ensure effective mixing [30]. Because of the effective mixing provided by the tesla valves that introduce turbulent flow and the length of the serpentine channels, our procedure for the special mixing chamber offers a reliable way to bind the target proteins to the antibody-conjugated beads.

### 3.3. Experimental Setup

**Figure 3:**
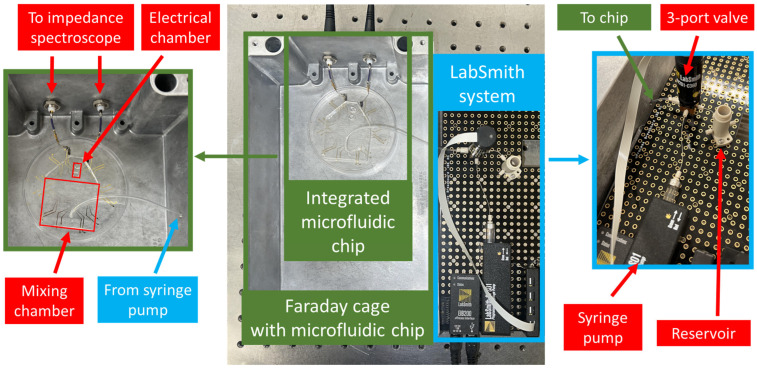
Experimental setup for the fully automated protein detection system. Experimental setup for the detection of protein-bound beads. The Faraday cage houses the microfluidic chip with integrated gold electrodes. The LabSmith fluid control system is depicted on the right. LabSmith system comprising of a programmable syringe pump, a 3-port valve, and a reservoir that interfaces to the microfluidic chip via a tube. Contents inside the Faraday cage are displayed on the left-hand side of the Faraday cage.

After confirming the reliability of the experimental procedure for binding the proteins to the antibody-conjugated beads, we moved on to the electrochemical measurement of the proteins using multi-frequency impedance spectroscopy. Figure 3 depicts the electrical protein detection system’s configuration. The Faraday cage used for minimizing the electrical interference presented to the electrical impedance spectroscopic measurements is shown on the left-hand side of Figure 3, whereas the automatic flow control system used for controlling and automating the fluid pumping steps can be seen on the right-hand side of the figure. A hole is drilled in the Faraday cage to allow a tube to pass through the structure for passing fluid to the integrated microfluidic chip with gold electrodes. Figure 3 depicts the fluid control system on the breadboard towards the right side. The fluid control system consists of a reservoir for holding the fluid, a syringe pump, and a 3-port valve. The 3-port valve connects the syringe pump to either the reservoir or the integrated microfluidic chip for the detection of proteins, depending on the configuration. The gold electrodes are connected to the impedance spectroscope through jumper wires connected to BNC cables, as shown in the figure.

### 3.4. Machine Learning Framework

**Figure 4:**
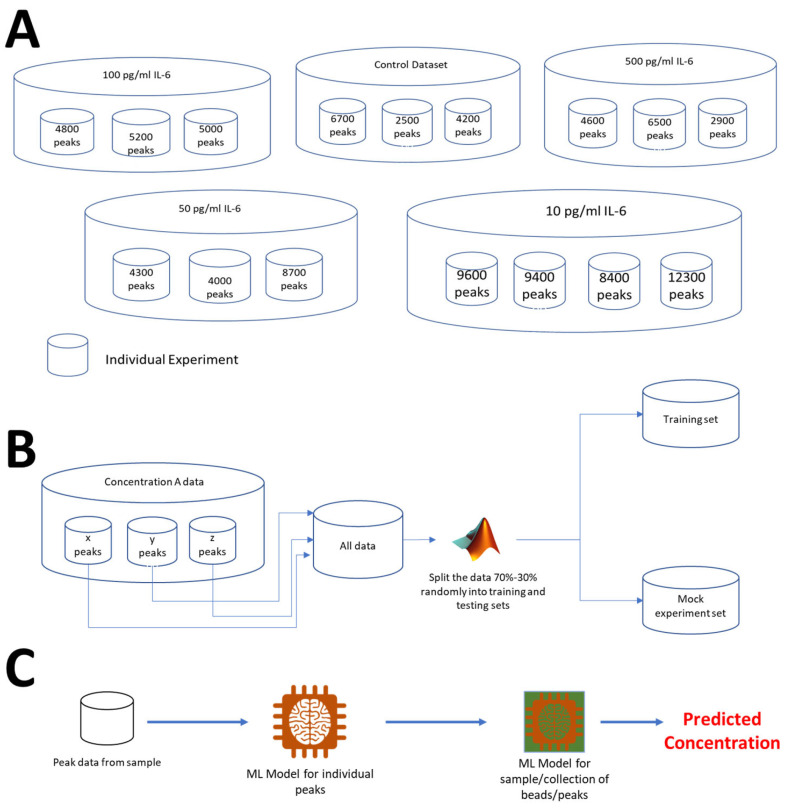
Overview of Machine Learning Framework. (**A**) A representation for each of the datasets. Each individual cylinder represents a single experiment carried out for 30 min. (**B**) Pooling and division of data into mock experiment set and the training set. (**C**) Two models are used for determining the concentration of a sample. The first model predicts the concentration for each individual peak in the form of a probability. The second model uses the probability for each of the beads in the sample from the first model as an input and predicts the concentration of a sample.

Figure 4A illustrates the dataset recorded for the protein IL-6. We recorded five different concentrations for the protein IL-6, namely, 10 pg/mL, 50 pg/mL, 100 pg/mL, 500 pg/mL, and the negative control dataset. Each peak corresponds to a bead passing through the gold electrodes. A typical raw modulated signal for beads passing through the microfluidic chip can be seen in our previous work [31]. A visual depiction of the dataset for a specific concentration is shown in Figure 4B. For each of the protein concentrations we tested, we performed at least three experiments, as shown in Figure 4B, where each small cylinder indicates a unique experiment made up of a specific number of peaks recorded as peak intensities at eight frequencies. As a result, each bead has eight data points, and the sample to be tested contains multiple beads to measure the protein concentration. The data is first pooled and then randomly divided into training and testing groups in a 70:30 ratio.

Since we further segment the testing set into mock experiments, which we use to imitate real experiments that are considerably shorter in length than the experiments used to train the model, the testing set is also known as the mock experiment set. This is done to test the model’s suitability for data gathered over a shorter time span and the reusability of the automated protein detection system when applied to the same chip. Our machine learning models now have two layers. Each peak in the machine learning model is categorized as corresponding to a specific protein concentration in the first layer. We apply a second layer of machine learning to this data set after this machine learning model has predicted the beads in each sample to correlate to a specific protein concentration based on the training data. This machine learning algorithm merely predicts the protein concentration of the entire sample. Figure 4C provides a visual representation of the system architecture.

### 3.5. Classification of Protein Concentrations

**Figure 5:**
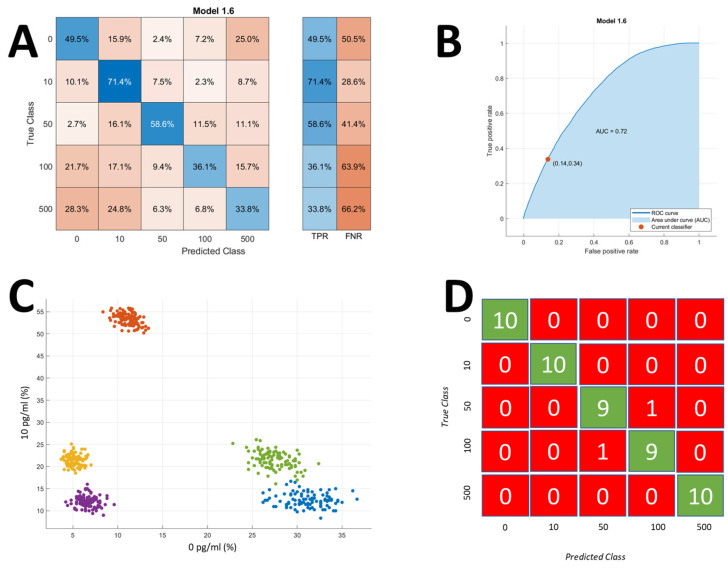
Predicted protein concentration using ML models. (**A**) Percentage of beads classified into a single concentration for 100 mock experiments. (**B**) The Area Under the Curve graph for the 10 pg/mL concentration of IL-6 experiments. (**C**) Training data are represented as a scatter plot of the percentage of beads classified into a single concentration for mock experiments. There are a total of 100 mock experiments for each concentration, which are color-coded—Blue: 0 pg/mL, Orange: 10 pg/mL, Yellow: 50 pg/mL, Purple: 100 pg/mL, Green: 500 pg/mL. (**D**) Confusion matrix for 50 mock experiments. This is the final confusion matrix representing the predicting and true class for sample concentration.

Here, we examine the data from the different Interleukin-6 (IL-6) protein concentrations in order to comprehend this idea. In order to categorize each individual bead into a protein concentration, we combine all the data from the three studies for the different protein concentrations. Figure 5A displays the confusion matrix for the individual beads. The rows of the confusion matrix correspond to the true class for the protein concentration, which is labeled as 0 for negative control, 10, 50, 100, and 500 for 10 pg/mL, 50 pg/mL, 100 pg/mL, and 500 pg/mL protein, respectively. The predicted class for each bead is shown in the columns. It was found that the coarse k-nearest neighbor algorithm provided the best results for the classification of beads with varying protein concentrations into their respective groups. It can be observed from the confusion matrix that the beads present in their true class correspond well with the predicted class, as the ratio of the predicted class to the true class is higher for the correct concentrations. As such, we utilize the results from this confusion matrix to train and classify a second layer of the ML algorithm to classify the sample, i.e., a collection of beads/peaks into their respective concentrations. Figure 5B shows the area under the curve for 10 pg/mL concentration data. The area under the curve is a depiction of the true positive rate vs. the false positive rate for each individual peak, which represents the classification of each single bead into a corresponding concentration. Although the classification accuracy for each individual peak is not acceptable, particularly for applications as sensitive as squamous cell carcinoma, we define a group of detected beads as a single mock experiment. In this case, the test sample now comprises 100 mock experiments for each concentration, where each mock experiment comprises a group of beads/peaks. The model we obtained in Figure 5A is applied to these mock experiments, and the percentage of the beads corresponding to each concentration is recorded in a matrix. The result of this matrix is shown as a scatter plot in Figure 5C. This plot shows the data as a scatter plot where the X-axis is the percentage of beads classified as belonging to the control group, i.e., concentration is 0 pg/mL, whereas the Y-axis of the plot corresponds to the percentage of beads classified as having a concentration of 10 pg/mL. We can see that, even with these two features, we are easily able to see a difference between the different concentrations of the proteins. We use the data obtained from these experiments to train the second layer of the ML model, which is a coarse k-nearest neighbor, which gives the concentration of each sample instead of each individual peak. In this case, the accuracy of the model is greatly improved by switching from trying to detect each individual peak and assigning it a group of either concentration to looking at the sample as a whole as a system in which beads are detected with varying probabilities of protein concentration and determining the characteristics of the sample as opposed to individual peaks. We then apply our developed ML model, which consists of two layers, to the test data, which we did not use for training these models. This test data is divided into mock experiments such that we only use 10% of the beads detected during a 30-min experiment. We divide the data into smaller pieces to show that the detection and assay time for the protein quantification can be reduced significantly and that we only used 30 min for gathering the impedance data so that we may have a dataset large enough to demonstrate the efficacy of our ML-assisted electronic protein quantification system. The accuracy of our system for the quantification of IL-6 protein in solution into various concentrations is 96%, as can be seen from the confusion matrix in Figure 5D. This confusion matrix shows the application of our developed ML models to a representative sample containing impedance data from beads that can be obtained in less than 3 min. A comparison of the different ML models applied using the MATLAB machine learning toolbox can be found in Table S1.

### 3.6. A Binary Classifier for OSCC

**Figure 6:**
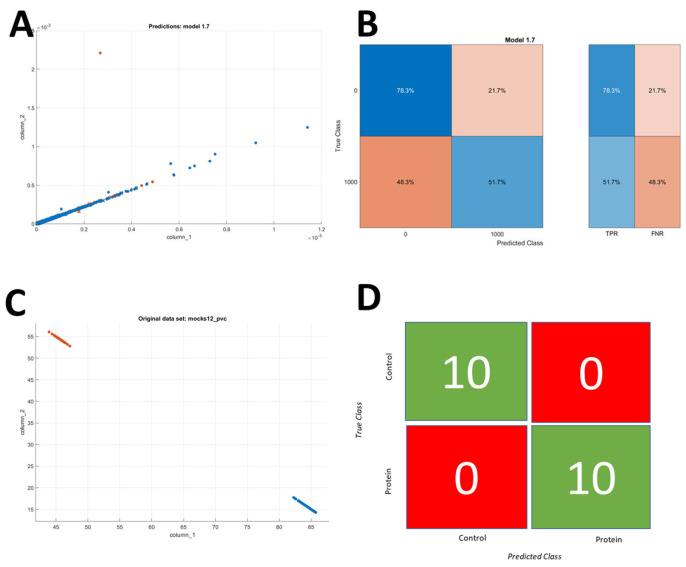
Data pooled from two chips for OSCC prediction. (**A**) Scatter plot showing the peak amplitude values (V) for two frequencies. The blue color represents the control group, whereas the orange color represents the protein. The X-axis shows the peak amplitude values at 500 kHz, and the Y-axis shows the peak amplitude at 700 kHz. (**B**) The linear discriminant model is applied on individual beads for training the first layer of the ML model. (**C**) The first layer is applied to 100 mock experiments from the pooled data. (**D**) Confusion matrix representing the developed ML model for the prediction of oral squamous cell carcinoma (OSCC) on test data divided into 10 mock experiments.

In order for sensitive applications such as the detection of squamous cell carcinoma, we need to develop a binary classifier, and 96% accuracy may be too low for such an application. Therefore, we use data pooled from two microfluidic chips, as well as the control data, and pool the other concentration data together into one category. The peaks are then randomly selected from this dataset to balance the size of the control and protein datasets. The dataset obtained from the second microfluidic chip can be seen in Figure S1. We obtained data for additional concentrations for the second microfluidic chip so that, in a future iteration, we can implement a linear regression model. However, the dataset from the additional concentrations, i.e., 25 pg/mL and 300 pg/mL, was not used in the current work. Figure 6A shows the scatter plot for the control dataset, and, at first glance, it seems like the dataset is identical for the two groups. However, when we implement a linear discriminant ML model on the dataset, we obtain the confusion matrix depicted in Figure 6B. In this figure, we can see that there is a clear difference in the probabilities for the two groups when the beads are labeled according to their particular concentration group, i.e., either protein or negative control. We then employ a similar method, such as the one used before, to implement the second layer of the ML model, where we use a coarse k-nearest neighbor algorithm for the implementation of the second layer, which deals with the whole sample instead of the individual peaks/beads. Figure 6C shows the first layer of the ML model applied to the 100 mock experiments, and we can see there is a clear difference between the two concentrations. We then use this developed ML model with two layers on the test data, which is divided into 10% of its original peaks, so that we can have detection from 3 min’s worth of experiments. These 10 mock test experiments were all classified into their respective groups, and we obtained a classification accuracy of 100%, as shown in Figure 6D. Therefore, we are able to successfully implement a robust fully automated method for the automated electronic detection of proteins.

## 4. Discussion

We have successfully demonstrated an automated method for the electronic detection of proteins in solution using multi-frequency impedance cytometry aided by machine learning and an automated fluid control system in a reusable microfluidic chip that has an integrated mixer and gold electrodes. Although we have demonstrated the ability of this system to detect and quantify protein in solution, there are some limitations. One such limitation of the current system is that we need to calibrate each individual chip using a negative control sample. This is a fairly simple fix, as each device can be calibrated before it is shipped for use. However, this poses a significant challenge as each device will need to have calibration data associated with it if such a device is to be used for a point-of-need-based solution in a resource-limited setting. Furthermore, the current system uses a commercial benchtop impedance spectroscope for implementing the lock-in amplification. This instrument is rather bulky and expensive, which is suitable for a proof-of-concept stage in the laboratory but will need to be replaced with a smaller, portable, and lower-cost readout system for use in a low-resource setting. Fortunately, there are many instances of portable and wearable lock-in amplifiers that have been developed and are prevalent in the literature [32,33,34]. Additionally, the data analysis is currently performed offline on a PC through MATLAB. We will need to perform the data analysis in real time on a portable computing device such as Raspberry Pi in the future. Therefore, in future work, we will perform the computations on a microcontroller system.

The accuracy of the current system for protein quantification is 96%. Although this accuracy might be acceptable in some scenarios, for certain applications where we want to have a precise measurement for protein quantification, we need a better model where we can accurately predict the concentration of the protein. Currently, we are classifying the sample into either concentration, but we are not predicting the precise concentration of the protein in a given sample. To achieve this, we need to develop a regression model for the concentrations and, therefore, we need more data points between the concentrations. As a result, we have obtained more data points for the concentrations for the experiments performed on a second chip (see Figure S1). We plan to populate the concentrations further and develop a regression model in a future iteration of this work. Another idea is to apply convolutional neural networks (CNNs) to the impedance data. For this purpose, we can convert each peak data into an image. This image will have 8 × 80 pixels, where 8 represents the number of frequencies and 80 corresponds to the number of samples in which a peak is typically present. The image data is explained in Figure S2. We can see a peak and its corresponding peak intensity plotted in grayscale in Figure S3. We also developed a simple neural network using MATLAB, which can be seen in Figure S4, and trained the image data obtained from the 50 pg/mL IL-6 using the developed neural network. The results for the validation and training can be seen in Figure S5. These are the preliminary results, and further work is still needed to optimize the neural network structure and training algorithm.

The ultimate goal of the developed system is to apply the proof-of-concept developed here and expand it to saliva samples that have been spiked with known levels of protein. If these experiments yield favorable results, we should move on to modify the system to be used for clinical samples and develop a database and ML models for use in an actual clinical setting. This work is a proof-of-concept stage and paves the way for these future steps to be taken towards realizing a fully automated point-of-need solution for the detection of various proteins and salivary biomarkers.

## Data Availability

Data available upon reasonable request.

## Supplementary Materials

The following supporting information can be downloaded at: https://www.mdpi.com/article/10.3390/s25051566/s1, Figure S1: Dataset obtained from experiments carried out for the second microfluidic chip; Figure S2: Impedance peak converted to a color image of 8 × 80 pixels where the 8 rows represent the frequency data whereas the columns represent the sample number. The pixel intensity gives the impedance peak amplitude.; Figure S3: Impedance peak converted to an image. The intensity of the image is plotted in grayscale. The left and right padding of the image is left out in this plot for clarity, however the image has a dimension of 8 × 80 to accommodate larger peaks so that the input to CNN may have a uniform structure; Figure S4: A simple neural network developed for the preliminary analysis of the images obtained from peak impedance data; Figure S5: Training the neural network on impedance data from 50 pg/mL IL-6 concentrations. Table S1: Comparison of various ML models to classify protein concentrations using MATLAB machine learning toolbox.
