# Supplementary material for: Automated Electrical Detection of Proteins for Oral Squamous Cell Carcinoma in an Integrated Microfluidic Chip Using Multi-Frequency Impedance Cytometry and Machine Learning
Source: Sensors (Basel). 2025 Mar 4;25(5):1566. doi: 10.3390/s25051566 (PMC11902334; doi:10.3390/s25051566)
Supplement: Supplementary file 1 [file sensors-25-01566-s001.zip › sensors-3334944-supplementary.pdf]

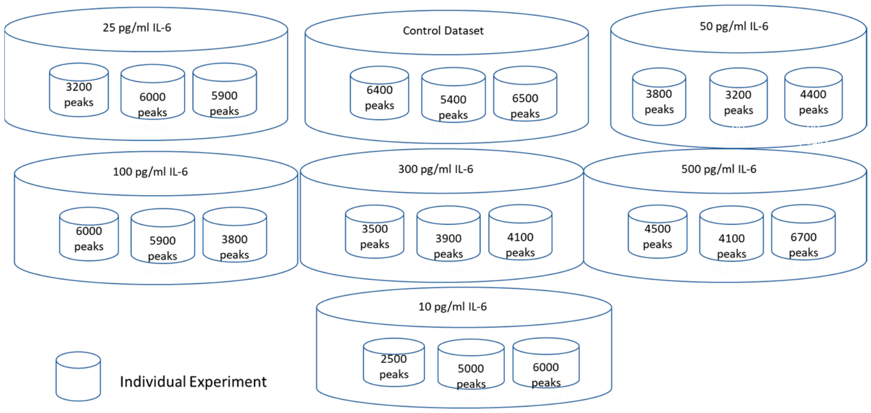

Figure S1 Dataset obtained from experiments carried out for the second microfluidic chip

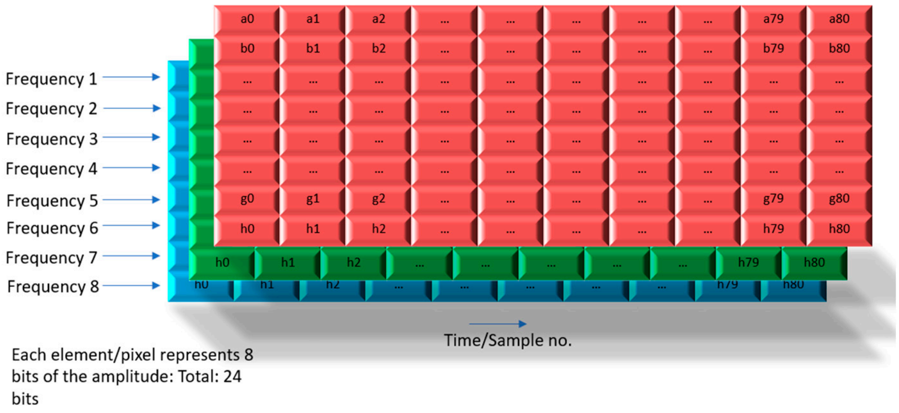

Figure S2 Impedance peak converted to a color image of 8 x 80 pixels where the 8 rows represent the frequency data whereas the columns represent the sample number. The pixel intensity gives the impedance peak amplitude.

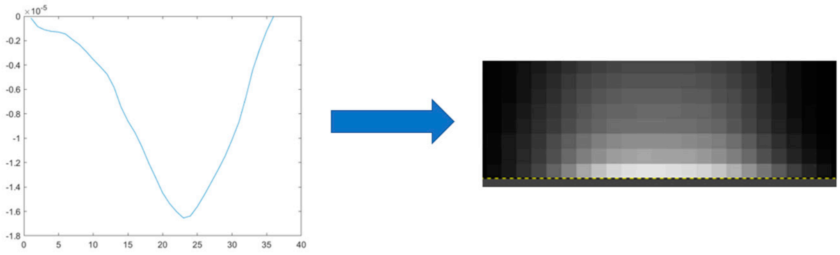

Figure S3 Impedance peak converted to an image. The intensity of the image is plotted in grayscale. The left and right padding of the image is left out in this plot for clarity, however the image has a dimension of 8 x 80 to accommodate larger peaks so that the input to CNN may have a uniform structure.

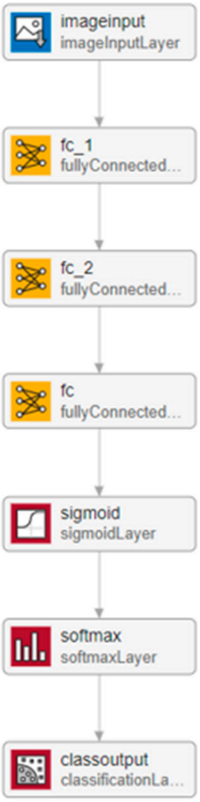

Figure S4 A simple neural network developed for the preliminary analysis of the images obtained from peak impedance data

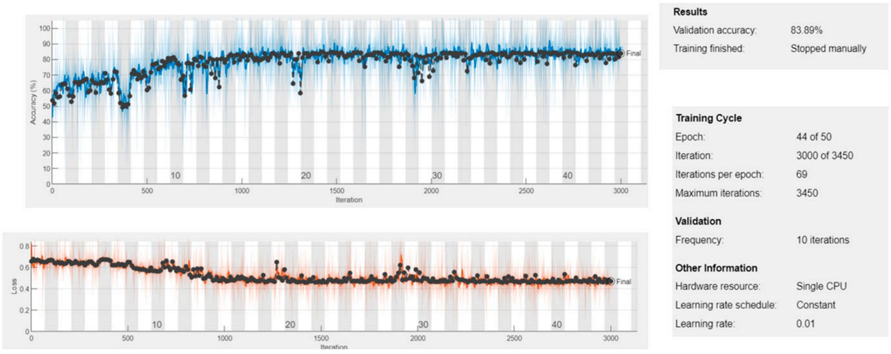

Figure S5 Training the neural network on impedance data from 50 pg/ml IL-6 concentrations.

Table S1. Comparison of various ML models to classify protein concentrations using MATLAB machine learning toolbox.

Method used for classification	Accuracy for classification(%)
**Coarse k-nearest neighbor algorithm**	**96**
**Cosine k-nearest neighbor algorithm**	**93**
**Coarse Tree**	**89**
**Medium Tree**	**91**
**Medium k-nearest neighbor algorithm**	**92**
